# Normal epithelial cells trigger EphA2-dependent RasV12 cell repulsion at the single cell level

**DOI:** 10.1080/21541248.2017.1324940

**Published:** 2017-06-21

**Authors:** William Hill, Catherine Hogan

**Affiliations:** European Cancer Stem Cell Research Institute, School of Biosciences, Cardiff University, Cardiff, UK

**Keywords:** cell repulsion, EphA2, epithelial, extrusion, RasV12, single cell

## Abstract

Epithelial cells expressing oncogenic Ras (RasV12) are detected by normal neighbors and are often extruded from tissues. We recently demonstrated that differential EphA2 signaling drives the segregation of mutant cells from normal monolayers via cell repulsion and increased RasV12 cell contractility. EphA2 signaling on RasV12 cells is triggered by ephrin-A ligands presented by normal cells. Here, we show that normal epithelial cells trigger the repulsion and enhanced contractility of Ras-transformed epithelial cells at the single cell level. We also reveal that ephrin-A ligands expressed on RasV12 cells are not required to drive RasV12 cell segregation following interaction with normal cells. Thus, normal-RasV12 cell-cell interaction triggers EphA2 forward signaling in RasV12 cells to drive repulsion and segregation of the transformed cells.

## Introduction

In epithelial tissues, transformed and normal cells compete for space and survival. This competitive process relies on the ability of a cell to detect changes in its neighbor, and results in the elimination of one cell population.^1^ Unsurprisingly, cell competition plays a role in quality control and homeostasis, and may also be tumor promoting or suppressive depending on the context and the genetic mutation expressed by the transformed cell.^2^ We, and others, have previously shown that epithelial cells expressing oncogenes such as RasV12 or v-Src are detected by normal neighbors and are eliminated by a process of extrusion.^11,3^ Oncogene-expressing cells are predominantly extruded apically, suggesting that this process may be a protective mechanism against tumor initiation.^5^ Several studies have detailed the mechanisms underlying RasV12 cell extrusion. This process requires E-cadherin-dependent cell-cell adhesion between RasV12 and normal cells, signaling to the actin-myosin cytoskeleton,^06,3^ as well as to intermediate filaments in normal cells.^7,8^ Moreover, downstream signals via Rho GTPases^9^ and Rab5-mediated endocytosis are also positive regulators of RasV12 cell extrusion.^10^ However, the upstream signal that triggers these events has remained elusive. We have recently revealed that epithelial cells detect and respond to neighboring cells overexpressing Eph receptors.^11^ As a result, the Eph overexpressing cell is triggered to segregate and extrude from normal tissues both in vitro and in vivo. EphA2 receptor tyrosine kinase is a transcriptional target of Ras-MAPK signaling^12^ and is expressed at elevated levels in epithelial cells expressing oncogenic RasV12 in a MEK-ERK-dependent manner.^11^ Our data demonstrates that enhanced expression of EphA2 in RasV12 cells promotes their detection by and separation from normal neighbors.^11^ Cell-cell interactions between normal and RasV12 cells induce EphA2 forward signaling on RasV12 cells in an ephrin-A ligand-dependent and E-cadherin-dependent manner. This triggers repulsion and an increase in cell contractility of RasV12 cells in direct contact with normal cells. In turn, neighboring RasV12 cells that are positioned behind marginal cells and not in direct contact with normal cells are triggered to contract in an EphA2-dependent manner. In this study, we further explore RasV12-normal cell-cell interactions to show that RasV12 cell repulsion and segregation from normal cells occurs at the single cell level, independent of ephrin-A ligands expressed on RasV12 cells.

## Results and discussion

To explore cell-cell interactions between Ras-transformed and normal epithelial cells, we use co-culture systems and Madin-Darby canine kidney (MDCK) epithelial cell lines, expressing GFP-tagged, constitutively active, oncogenic Ras (RasV12) in a tetracycline/doxycycline-inducible manner.^3,11^ Using these lines we generate mosaic epithelial cell sheets by mixing RasV12 cells with normal cells at 1:100 ratios in the absence of tetracycline.^3,11^ Once cell-cell adhesion is established and an epithelial monolayer has formed, tetracycline is added to the cells and GFP-RasV12 expression is induced. More recently, we have developed the cell confrontation assay, which allows collision between sheets of RasV12-expressing and normal cells.^11^ Using both assays we have demonstrated that interaction with normal cells triggers RasV12 cells to become round and contractile, and to segregate away from the normal cells. When present as single cells or small clusters within normal monolayers, RasV12 cells are eventually apically extruded from the tissue. In cell confrontation assays, collision with normal cells triggers a rapid cell repulsion of RasV12 cells; cells stop migrating forward and actively migrate backward.^11^ In addition, normal cell sheets continued to migrate forward with intermingling between the two populations of cells significantly inhibited. We have previously shown that segregation of RasV12 cells from normal cells is driven by an EphA2-dependent cell repulsion.^11^ Moreover, neighboring RasV12 cells positioned behind the marginal cells that are not in direct contact with the normal cells also contract and round up in an EphA2-dependent manner.^11^ However, we could not conclusively determine whether this was a ligand-dependent process. Moreover, when using the confrontation assay, we were also unable to conclusively determine whether normal cell sheets, which migrate forward, were physically compressing the RasV12 cells backward.

**Figure 1.**
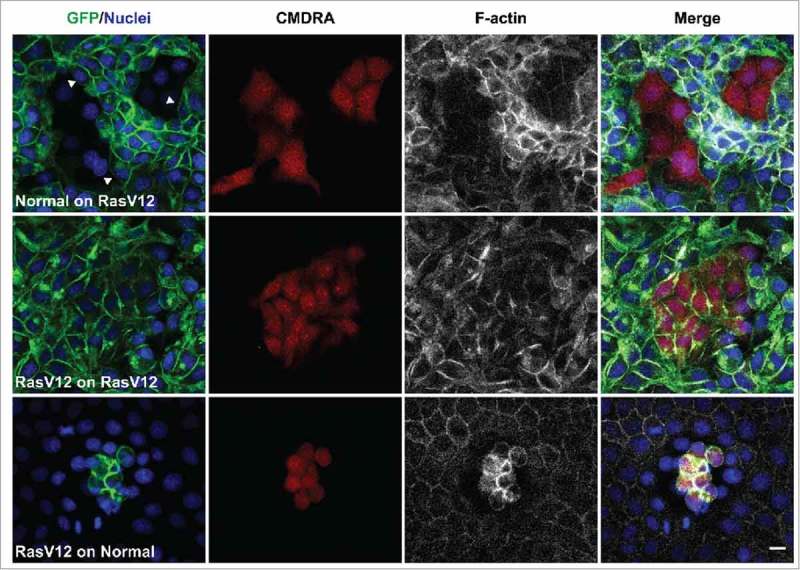
Normal cells induce RasV12 cell repulsion and increased contractility at the single cell level. Confocal images of coculture assays. Upper panels: Normal MDCK cells prelabeled with cell tracker dye (CMDRA) are added to confluent monolayers of GFP-RasV12 cells. Middle panels: GFP-RasV12 cells prelabeled with cell tracker dye (CMDRA) are added to confluent monolayers of GFP-RasV12 cells. Lower panels: GFP-RasV12 cells prelabeled with cell tracker dye (CMDRA) are added to confluent monolayers of normal MDCK cells. Cells were fixed 24 h after addition of prelabeled cells and stained with phalloidin (gray) and Hoechst (blue). Scale bar, 20 μm.

To explore each of these points further, we used our MDCK cell systems to investigate whether normal cells could induce RasV12 cell repulsion and changes in RasV12 cell contractility when co-cultured with RasV12 cells at low densities. To monitor cell repulsion, we first seeded GFP-RasV12 cells as confluent monolayers in the presence of tetracycline, before adding normal cells (pre-labeled with cell tracker dye) to the apical side of the monolayers at low densities. In parallel, we added labeled GFP-RasV12 cells at low densities to the apical side of confluent normal monolayers, or to confluent monolayers of GFP-RasV12 cells. Cells were fixed 24 h later and monitored by immunofluorescence. We found that labeled normal cells flattened and spread with a regular morphology in between GFP-RasV12 cells (Fig. 1). RasV12 cells juxtaposed to the normal cell cluster appeared round and contractile, and F-actin accumulated at cell-cell contacts between the contractile RasV12 cells (Fig. 1). RasV12 cell repulsion was evident at the interface with the cluster of normal cells, as GFP-RasV12 cell membranes appeared smooth and free of protrusions, and cells often aligned perpendicular to the normal cell cluster (Fig. 1, arrowheads). Labeled GFP-RasV12 cells dropped on top of GFP-RasV12 cells flattened, spread and formed homogeneous cell-cell adhesions with unlabelled neighbors (Fig. 1). In contrast, GFP-RasV12 cells dropped onto confluent monolayers of normal cells did not spread or intercalate with the normal cells, but remained on top of the normal cells as tight clusters of contractile cells (Fig. 1), similar to previous reports.^3^ Together, these data suggest that even when present at low numbers, normal cells can induce RasV12 cell repulsion and increased RasV12 cell contractility.

**Figure 2.**
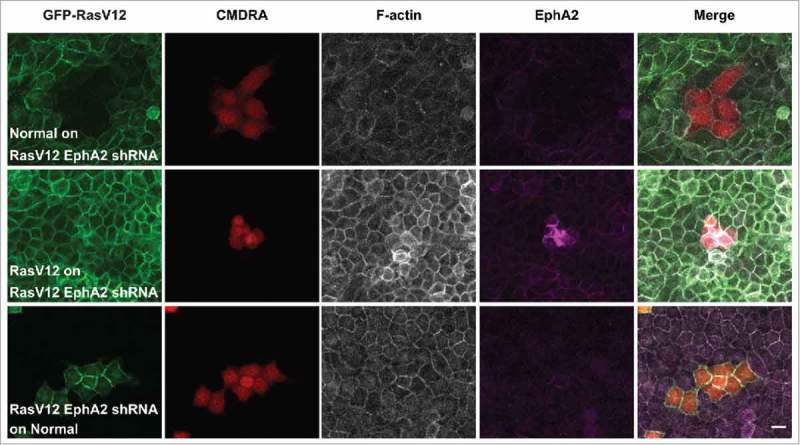
EphA2 receptor expressed on RasV12 cells is required to drive RasV12 cell repulsion and contractility following interaction with normal cells. Confocal images of coculture assays. Upper panels: Normal MDCK cells prelabeled with cell tracker dye (CMDRA) are added to confluent monolayers of GFP-RasV12 cells constitutively expressing EphA2 shRNA. Middle panels: GFP-RasV12 cells prelabeled with cell tracker dye (CMDRA) are added to confluent monolayers of GFP-RasV12 cells constitutively expressing EphA2 shRNA. Lower panels: GFP-RasV12 cells constitutively expressing EphA2 shRNA prelabeled with cell tracker dye (CMDRA) are added to confluent monolayers of normal MDCK cells. Cells were fixed 24 h after addition of prelabeled cells and stained with phalloidin (gray), anti-EphA2 antibody (magenta) and Hoechst (blue). XY images represent maximum projections of z-stacks. Scale bar, 20 μm.

We next tested the functional role of EphA2 in this assay, and used GFP-RasV12 cell lines that constitutively expressed EphA2 shRNA as described previously.^11^ When labeled normal cells were dropped on to monolayers of RasV12 depleted of EphA2, we did not observe an increase in RasV12 cell repulsion and contractility (Fig. 2). Here, RasV12 cells appeared regular in shape and formed homogeneous cell-cell adhesions with the normal cells. Moreover, when dropped onto normal monolayers, RasV12 cells depleted of EphA2 spread and formed cell-cell adhesions with normal cells (Fig. 2). We observed that labeled RasV12 cells added to monolayers of RasV12 cells depleted of EphA2 remained as contractile clusters of cells, on the apical side of the monolayer (Fig. 2). This was not surprising since RasV12 cells constitutively expressing EphA2 shRNA behave like normal cells in coculture assays.^11^ Together these data support our previous findings that EphA2 expressed on RasV12 is required to drive RasV12 cell repulsion and contraction following interaction with normal cells.

**Figure 3.**
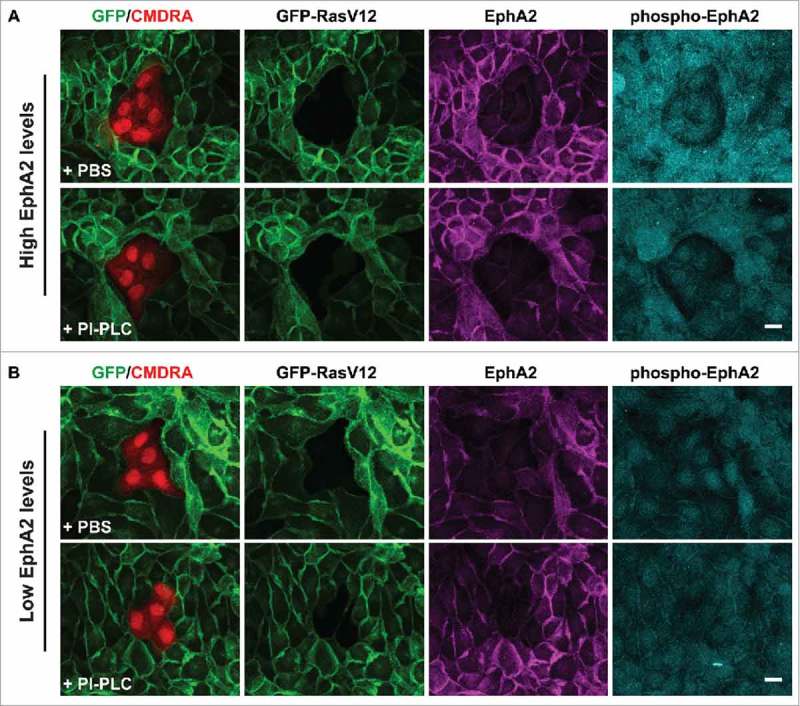
RasV12 cell repulsion and contractility is dependent on endogenous EphA2 expression levels and not on coexpressed ephrin-(A)ligands. Confocal images of coculture assays. (A) Normal MDCK cells prelabeled with cell tracker dye (CMDRA) are added to confluent monolayers of GFP-RasV12 cells that expressed high levels of EphA2 and were pretreated with either PBS (upper panels) or phosphatidylinositol-specific phospholipase C (PI-PLC) (lower panels). (B) Normal MDCK cells prelabeled with cell tracker dye (CMDRA) are added to confluent monolayers of GFP-RasV12 cells that expressed low levels of EphA2 and were pretreated with either PBS (upper panels) or phosphatidylinositol-specific phospholipase C (PI-PLC) (lower panels). Cells were fixed 24 h after addition of prelabelled cells and stained with anti-EphA2 (magenta), anti-phosphorylated EphA2 (Y594) (phospho-EphA2; cyan) and Hoechst (blue). XY images represent maximum projections of z-stacks. Scale bar, 20 μm.

In our previous study, we demonstrated that removal of ephrin-A-ligands from the cell surface of non-transformed cells, or depletion of EphA2 expressed on RasV12 cells promoted intermingling of RasV12 cells with non-transformed cells, the loss of RasV12 cell contractility, and the formation of basal protrusions. Moreover, preclustered ephrin-A ligands triggered cell repulsion, or clustering of RasV12 cells in the absence of non-transformed cells. Thus, our data suggested that forward signaling via EphA2 receptor expressed on RasV12 cells is sufficient to induce contractility and clustering of RasV12 cells following interaction with non-transformed cells. RasV12 cells express ephrin-A1 and -A4 mRNA at levels equivalent to normal cells.^11^ Therefore, we wanted to explore whether ephrin-A ligands expressed on RasV12 cells are also required to drive RasV12 cell contractility and segregation. We reasoned that by using this new assay (as described above) we could explore the propagation of an Eph-ephrin signal between neighboring RasV12 cells at the single cell level. We first removed GPI-linked ephrin-A ligands from the cell surface of RasV12 cells by treating the cells with the enzyme phosphatidylinositol-specific phospholipase C (PI-PLC) to remove endogenous ephrin-A ligands from RasV12 cells.^11^ We seeded PI-PLC treated (or PBS-treated) GFP-RasV12 cells as confluent monolayers before adding pre-labeled normal cells on top of the monolayers at low density. Cells were fixed and stained 24 h later. We found that normal cells flattened and spread into monolayers of both PI-PLC treated and PBS treated RasV12 cells (Fig. 3). However, we found that RasV12 cell responses were dependent on the levels of EphA2 expressed on RasV12 cells, irrespective of treatment. When the level of EphA2 expressed on RasV12 cells was high, we observed cell repulsion in both PBS and PI-PLC treated RasV12 cells in direct contact with normal cells. Moreover, RasV12 cells appeared round and contractile in both PBS and PI-PLC treated monolayers. Using phospho-specific antibodies to monitor Eph receptor clustering and activation,^13^ we detected elevated levels of phosphorylated EphA2 (Y594) in RasV12 cells expressing high levels of EphA2 and surrounding a normal cell cluster (Fig. 3). In contrast, RasV12 cells expressing low levels of EphA2 showed low levels of cell repulsion and were not contractile following interaction with normal cells. Here, we detected low levels of phosphorylated EphA2. Together, these data suggest that ephrin-A ligands expressed on RasV12 cells are not required to induce contractility and repulsion between neighboring RasV12 cells following interaction with normal cells. These data also support our previous findings that differential EphA2 expression and hence signaling drives repulsion and segregation of cells expressing elevated levels of EphA2 receptor.

In summary, we have shown that normal epithelial cells trigger the repulsion and enhanced contractility of Ras-transformed epithelial cells at the single cell level. Using a new coculture assay, we have demonstrated that even small clusters of normal cells can induce these responses in confluent monolayers of RasV12 cells. Thus, normal-RasV12 cell-cell interaction triggers a signaling event rather than normal cells compress RasV12 cells. We also found that ephrin-A ligands expressed on RasV12 cells do not play a role in RasV12 cell repulsion and contractility following interaction with normal cells. In our previous report we showed that RasV12 cells positioned behind the marginal cells and not in direct contact with normal cells are also triggered to contract in an EphA2-dependent manner.^11^ Our data presented here suggest that signaling from marginal RasV12 cells to neighboring RasV12 cells, occurs in an ephrin-A ligand-independent manner. It is possible that EphA2 when present at elevated levels on RasV12 cell membranes becomes mobile and forms higher order clusters, promoting autophosphorylation events.^14,15^ Activation of EphA2 in RasV12 cells drives RasV12 cell repulsion and changes in cell contractility in a myosin-II-dependent manner.^11^ Together with our previous findings, we conclude that cells detect and respond to steep differences in EphA2 expressed on neighboring cells and it is this differential EphA2 signal that drives the repulsion and segregation of the EphA2 overexpressing cell.

## Materials and methods

### Antibodies and reagents

Primary antibodies used: mouse anti-EphA2 (clone D7) (Millipore); rabbit anti-phospho-EphA2 (Y594) (p-EphA2). Secondary antibodies (Alexa-405- and Alexa-647-conjugated anti-mouse and anti-rabbit) were from Life Technologies. Orange cell tracker dye (CMDRA) and phosphatidylinositol-specific phospholipase C (PI-PLC) were both from Life Technologies.

### Cell culture and coculture assay

Non-transformed MDCK cells, MDCK-pTR cell lines stably expressing GFP-RasV12 in a Tet-ON inducible manner or MDCK-pTR GFP-RasV12 expressing EphA2 shRNA were cultured as described previously.^3,11^ MDCK-pTR-GFP-RasV12, non-transformed MDCK or MDCK-pTR-GFP-RasV12 EphA2 shRNA cells were trypsinised and seeded on serum-coated 18mm^2^ glass coverslips, at a density of 1 × 10^6^ cells. Cells were incubated for 8–16 h at 37^o^C to allow the formation of a confluent monolayer. MDCK-pTR-GFP-RasV12 cell lines were cultured in the presence of tetracycline/doxycycline to induce GFP-RasV12 expression. The next day, non-transformed (normal) MDCK, or MDCK-pTR GFP-RasV12 cell lines pre-labeled with cell tracker dye (CMDRA) were dropped onto the monolayers at a density of 2 × 10^3^. Cells were incubated for a further 24 h before fixation.

### Immunofluorescence

Immunofluorescence of cells cultured on serum-coated glass coverslips was performed as described previously.^3,11^ Anti-EphA2 antibody was used at a dilution of 1:100; anti-phosphorylated-EphA2 antibody was used at 1:50. All secondary antibodies were used at 1:200. TRITC-phalloidin (Sigma-Aldrich) was used at 1.5 μg ml^−1^. Cells cultured on glass coverslips were examined using a Zeiss LSM710 confocal microscope. Images were analyzed using Zen (Zeiss Efficient Navigation) software, or Fiji (National Institute of Health) (v1.48j) software.

### PI-PLC treatment

Endogenous ephrin-A ligands were cleaved from the cell surface of non-transformed cells as described previously.^11,16^ Briefly, cells were pre-treated with 1 U ml^−1^ PI-PLC or an equivalent volume of PBS for 4 h before being switched back to regular DMEM/FCS media. Cells were then trypsinised and seeded on serum-coated coverslips at a density of 1 × 10^6^. Cell lysates were also harvested in RIPA buffer at 4 h or 28 h post-treatment. Immunoblotting analyses were performed using the following antibodies: mouse anti-GAPDH at 1:2,000, rabbit anti-ephrin-A1 at 1:500.
